# Pattern and determinants of contraceptive usage among women of reproductive age from the Digo community residing in Kwale, Kenya: results from a cross-sectional household survey

**DOI:** 10.1186/s12905-017-0497-5

**Published:** 2018-01-08

**Authors:** Vernon Mochache, Amyn Lakhani, Hajara El-Busaidy, Marleen Temmerman, Peter Gichangi

**Affiliations:** 1 International Centre for Reproductive Health, Mombasa, Kenya; 2 University of Ghent, Ghent, Belgium; 3 Faculty of Health Sciences, Aga Khan University, Nairobi, Kenya; 4 Department of Health, County Government of Kwale, Kwale, Kenya; 5 University of Nairobi, Nairobi, Kenya; 6 P.O. Box 3921, Nakuru, 20100 Kenya

**Keywords:** Contraception, Uptake, Utilization, Unmet need, Determinants, Demand-side, Kwale, Digo, Kenya

## Abstract

**Background:**

Contraceptive usage has been associated with improved maternal and child health (MCH) outcomes. Despite significant resources being allocated to programs, there has been sub-optimal uptake of contraception, especially in the developing world. It is important therefore, to granulate factors that determine uptake and utilization of contraceptive services so as to inform effective programming.

**Methods:**

Between March and December 2015, we conducted a cross-sectional survey among women of reproductive age (WRA) from the Digo community residing in Kwale County, Kenya. The study aimed to describe the pattern and determinants of contraceptive usage in this population. Respondents were selected using stratified, systematic sampling and completed a household sexual and reproductive health (SRH) questionnaire.

**Results:**

We interviewed 745 respondents from 15 villages in 2 out of 4 sub-counties of Kwale. Their median (interquartile range, IQR) age was 29 (23–37) years. 568 (76%) reported being currently in a marital union. Among these, 308 (54%) were using a contraceptive method. The total unmet need, unmet need for spacing and for limiting was 16%, 8% and 8%, respectively. Determinants of contraceptive usage were education [adjusted Odds Ratio, aOR = 2.1, 95% confidence interval, CI: 1.4–3.4, *P* = 0.001]; having children [aOR = 5.0, 95% CI: 1.7–15.0, *P* = 0.004]; having attended antenatal care (ANC) at last delivery [aOR = 4.0, 95% CI: 1.1–14.8, *P* = 0.04] as well as intention to stop or delay future birth [aOR = 6.7, 95% CI: 3.3–13.8, *P* < 0.0001].

**Conclusions:**

We found high levels of contraceptive usage among WRA from the Digo community residing in Kwale. To further improve uptake and utilization of contraception in this setting, programs should address demand-side factors including ensuring female educational attainment as well as promotion of ANC and skilled birth attendance.

**Electronic supplementary material:**

The online version of this article (10.1186/s12905-017-0497-5) contains supplementary material, which is available to authorized users.

## Background

Contraceptive utilization has been shown to have both direct and indirect benefits on maternal and newborn health especially involving the prevention of pregnancies that pose a risk to maternal and child survival. Maternal and newborn deaths are averted by preventing pregnancy-associated complications; lowering the risk of unsafe abortions due to unwanted pregnancies; delaying first pregnancy among adolescent girls as well as reducing hazards related to high parity among older women [1, 2]. It has been shown that providing access to contraception for all women in developing countries where contraceptive uptake is currently low, could prevent up to 54 million unintended pregnancies, including 21 million unplanned births; 26 million abortions out of which 16 million would be unsafe; 7 million miscarriages; 79,000 maternal deaths as well as 1.1 million infant deaths [3].

Additionally, contraceptive usage has been shown to play a key role in the achievement of sustainable development goals (SDGs) through its impact on individuals, families, communities and nations [4–6]. These benefits encompass the promotion of gender equality and empowerment; poverty reduction and achievement of economic growth; contribution to peace and political stability; mitigation of the effects of rapid population growth on access to clean water and sanitation; expansion of access to clean and renewable energy; development of safe, resilient and sustainable cities as well as addressing challenges of climate change by mitigating the effects of deforestation and human-wildlife conflict. In this regard, expanding access to contraception is critical to achieving the post-2015 development agenda across the 5 SDGs themes of people, planet, prosperity, peace and partnerships.

To this end, significant investments have been made towards improving the uptake and utilization of contraception globally. At the 2012 London Summit on Family Planning, more than 20 governments made commitments to address the policy, financing and socio-cultural barriers to women accessing contraception worldwide [7]. To actualize these commitments, donors pledged an additional US$2.6 billion in funding to enable 120 million more women and girls to access and utilize contraception by 2020. Other investments have come through the United Nations Commission on Life-Saving Commodities for Women and Children [8] as well as the recently-launched *She Decides Initiative* that has raised more than €181 million for contraceptive programs globally [9].

So far, these investments have seemingly yielded positive gains, with some regions in Asia (excluding China), Latin America and the Caribbean reporting high contraceptive usage with attendant decline in total fertility rate (TFR). Unfortunately, regions in sub-Saharan Africa have continued to lag behind [10, 11]. The United Nations estimates that among the major geographic regions of the world, Africa still retains the highest annual population growth rate (2.55%) and this is projected to remain high in 27 sub-Saharan African countries. Further, Africa is expected to contribute more than half of global population growth by the year 2050 and as a result, of the additional 2.4 billion people projected to be added to the world’s population between 2015 and 2050, 1.3 billion will be in Africa [12].

Overall, Kenya has seen significant improvements in the uptake and utilization of contraception over the last three decades [13]. The contraceptive prevalence rate (CPR), which is the proportion of married women currently using a contraceptive method, has more than doubled from 27% in 1989 to 58% in 2014. Conversely, the proportion of women who want to postpone their next birth for two or more years or who want to stop childbearing altogether but are not using a contraceptive method (unmet need) has nearly halved from 35% in 1993 to 18% in 2014 [14]. Despite these reported improvements, there still remain disparities within specific regions and populations. For example, the unmet need is highest among women residing in rural areas, those without formal education and those in the lower wealth quintiles. Unmet need is also higher than the national average in the former North Eastern, Nyanza, Coast, Rift Valley and Western provinces.

Kwale County in the former Coast province is one such case-study. Located on the southern coastal strip of Kenya between Mombasa and the border with Tanzania, the county has a population of nearly 650,000 with a preponderance of females (51%). Residents of this county predominantly profess the Islamic faith (80%) and are mainly from the Digo community (80%). The county ranks among the poorest in Kenya (75% poverty rate) with a predominantly rural population (20% urbanization rate) [15–17]. Additionally, only 6% of Kwale County’s population reports having more than eight years of education. Against this backdrop, the current TFR of 4.7 is higher than the national average of 3.9 children per woman while the current CPR of 42% is lower than the national rate [18].

Levels of knowledge and uptake of contraceptive services in Kwale County have previously been reported as low [19]. In one such study, 61% of married women reported not using any form of contraception. Of these, only 2% gave health system barriers as the main reason for non-use. Other reasons cited included the experience and fear of side effects (11% and 21%, respectively), socio-cultural factors including those related to spousal or in-law objection (14% and 3%, respectively) as well as religious reasons (4%). About a third (29%) of the women interviewed cited other undefined reasons for non-use.

For this reason, several MCH programs have been implemented in Kwale County over the past five years with one objective being to improve uptake and utilization of contraceptive services [19–21]. The Missed Opportunities in Maternal and Infant (MOMI) health project was a five-year, multi-country, operations research initiative whose overall objective was to reduce maternal and newborn morbidity and mortality by taking advantage of missed opportunities in the postpartum period through a combined facility and community-based strategy. The project aimed to improve delivery of postpartum services, including contraception. The Mama Na Mtoto (MNM) II project on the other hand, was the second phase of a four-year, maternal and child health initiative with the aim of contributing towards a reduction in maternal and childhood mortality by targeting demand-creation in the community through capacity building of community health systems [21].

With this background, we sought to develop a better understanding of the pattern of contraceptive usage and the factors that determine contraceptive uptake and utilization in this setting. This will provide a basis for developing evidence-based programs that are responsive to local socio-cultural perspectives. It has been shown that SRH-related attitudes and behaviors could be influenced by societal norms, traditions, beliefs and cultural practices [22–24]. A better understanding of these factors will therefore provide an efficient framework for developing sustainable interventions that are not only effective, but also readily acceptable given their responsiveness to the local context.

### Study objectives and Aims

The general objective of this study was to determine the pattern and determinants of contraceptive usage among WRA from the Digo community residing in Kwale County, Kenya. Specifically, the study aimed to:

- Determine the unmet need for contraception among WRA from the Digo community residing in Kwale County, Kenya.
- Determine factors associated with utilization of contraceptive services among WRA from the Digo community residing in Kwale County, Kenya
- Develop recommendations for improving uptake and utilization of contraceptive services in this setting

## Methods

### Study design and setting

This was a cross-sectional household survey conducted between March and December 2015 within the framework of two MCH projects funded by the European Commission and implemented in 2 out of 4 sub-counties of Kwale County. The MOMI project was implemented in Matuga sub-county while the MNM II project was implemented initially in Msambweni and later, in Lungalunga sub-counties.

### Sample size and sampling procedures

Data collection involved administration of a structured SRH questionnaire to female respondents aged 18–45 years in their households (Additional file 1). We estimated a sample size of 700 respondents based on a previously reported CPR of 30% in this setting; a sample design effect of 2.5; *Z*-statistic of 1.96 for a 95% confidence level in the estimation; 10% non-response rate and a 5% margin of error. Respondents were selected using stratified, systematic random sampling. Each sub-county was stratified into constituent wards, sub-locations and further into villages within each sub-location.

From each village, we obtained a list of all households from the headman and randomly selected households to visit using a random number generator. The number of households selected was based on the proportion of households in that village relative to the total number of households in each sub-location with a sampling interval of 12 households. In each household, we administered the questionnaire to all female respondents who reported being from the Digo community and who were resident in the study area for more than 6 months. We excluded those who did not provide consent, those who were not resident in the study area and women aged <18 (unless they were emancipated minors) or >45 years old.

### Community engagement and ethical considerations

Prior to any data collection activities, we held a series of meetings with community gatekeepers including religious leaders, village headmen, chiefs, sub-county commissioners and the Kwale county commissioner. This was meant to sensitize them on the proposed data collection procedures and obtain their buy-in. We also used this as an opportunity to introduce our study team consisting of the principal investigator and resident data enumerators who were from the Digo community but residing in different villages within the study area.

Ethical approval for the study was obtained from the Research Ethics Committee of the Aga Khan University, Nairobi (2014/REC-51) and the Ethics Review Committee of the University of Nairobi and Kenyatta National Hospital (P502/08/2014). We also obtained a research permit from the National Commission for Science, Technology and Innovation (#4703) to facilitate the conduct of research activities in the community. All participants provided written informed consent.

### Data management and statistical analyses

Quantitative data were entered into a Microsoft Access (2010) database (Microsoft Inc. Seattle, WA, USA) and after appropriate data cleaning checks, migrated to Stata version 12 (StataCorp Inc., College Station, TX, USA) for statistical analyses. For the descriptive statistics, we summarized the respondents’ demographic characteristics as counts (N) and percentages (%) for categorical data and median (IQR) for continuous data. We compared these characteristics using Pearson’s chi-squared test for categorical data and Wilcoxon rank-sum test for continuous data. For the inferential statistics, the outcome of interest was current usage of any contraceptive method. We calculated the odds of current contraceptive usage among women with each determinant of interest versus the reference group using multivariable logistic regression models and report the adjusted ORs and 95% CIs for each. All statistical tests were evaluated using a 2-sided α-value of 0.05.

## Results

Between March and December 2015, 10 resident data enumerators visited a total of 740 households in 15 villages from 2 out of the 4 sub-counties of Kwale County namely, Lungalunga and Matuga. In Lungalunga sub-county, we collected data from households in 12 villages within Pongwe/Kikoneni Ward among 2 sub-locations namely, Bumbani and Majoreni. In Matuga sub-county, we collected data from households in 3 villages within Tiwi Ward among 2 sub-locations namely, Simkumbe and Mkoyo. The median (IQR) age of the data enumerators was 37 (30–44) and 6 were female.

The enumerators administered the SRH questionnaire to a total of 745 female respondents, allowing for more than one eligible respondent per household. The median (IQR) age of the respondents was 29 (23–37). 568 (76%) were currently in a marital union i.e. either legally married or living with a man as if married while 646 (87%) reported they had ever attended school with the median (IQR) years of education being 8 (7–11). The reported median (IQR) ages at sexual debut and marriage were 18 (16–20) and 20 (18–23), respectively (Table 1). 632 (85%) respondents reported that they had ever given birth with the median (IQR) number of total births reported being 4 (2–5). Of these, 373 (59%) reported that they had intended to get pregnant at the time that they did.

**Table 1:** Demographic characteristics of survey respondents (*n* = 745)

Characteristic	N(%)/Median (IQR)
Respondent’s age	29 (23–37)
Husband/partner’s age	39 (30–46)
Age at sexual debut	18 (16–20)
Age at marriage/union	20 (18–23)
Marital status	
Currently married	426 (57%)
Currently living as if married	142 (19%)
Currently not in a union	177 (24%)
Ever attended school	646 (87%)
Years of education	8 (7–11)
Ever given birth	632 (85%)
Total number of births	4 (2–5)
Currently pregnant	75 (10%)
Duration (months) of current pregnancy	6 (4–7)

Of those respondents who reported they had not intended to get pregnant at the time that they did, 227 (89%) wanted to give birth later with a median (IQR) duration of 5 (3–5) years before the next pregnancy. Only 29 (11%) respondents reported that they had not wanted any more children at the time of their previous birth. Of the respondents who reported a previous birth, the median (IQR) number of children desired in the woman’s lifetime was 5 (4–6). 556 (75%) respondents said that they would want an additional child in the future. Of these, 468 (84%) said that they would like to wait for several years before having the additional child with a median duration of 5 (3–5) years before the next pregnancy. Sixty two (11%) respondents said that they would want an additional child as soon as possible. No respondent reported not wanting any children in her lifetime.

**Fig. 1:**
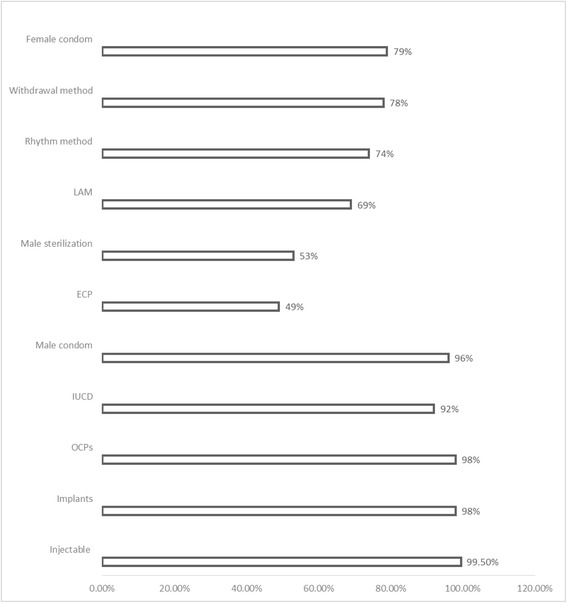
Knowledge of contraception among WRA from the Digo community residing in Kwale County, Kenya (*n* = 745)

With regards to knowledge of different contraceptive methods (Fig. 1), 380 (51%) respondents reported that they did not know about emergency contraception; 350 (47%) did not know about male sterilization; 233 (31%) did not know about lactational amenorrhea (LAM); 192 (26%) did not know about rhythm method while 161 (22%) and 154 (21%) did not know about withdrawal method and female condoms, respectively. Majority of respondents reported knowledge of injectable Depo-Provera (99.5%), contraceptive implants (98%), oral contraceptive pills (OCPs) (98%), male condoms (96%) and intrauterine devices (IUCDs) (92%).

**Fig. 2:**
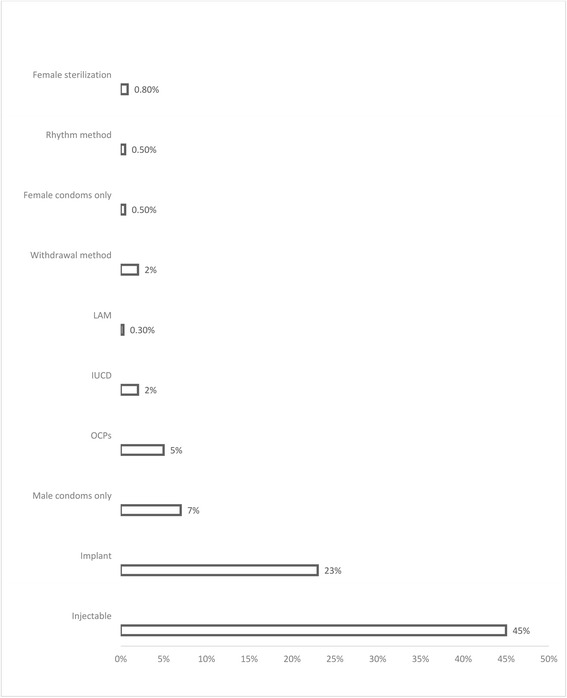
Contraceptive use among WRA from the Digo community residing in Kwale County, Kenya (*n* = 384)

With regards to contraceptive usage (Fig. 2), 386 (52%) respondents reported currently using a contraceptive method. Of these, 172 (45%) were using injectable Depo-Provera, 87 (23%) were using contraceptive implants, 25 (7%) reported using only male condoms while 19 (5%) were using OCPs. Only 7 (2%) respondents reported using an IUCD. Specifically, among women who were currently in a marital union, 308 (54%) reported current contraceptive usage.

**Fig. 3:**
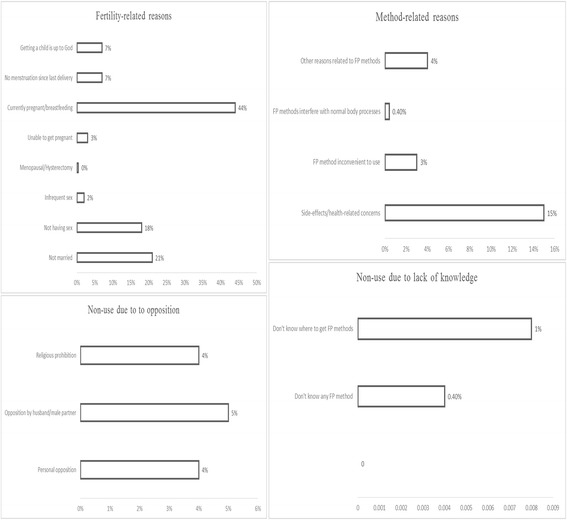
Reasons for contraceptive non-use among WRA from the Digo community residing in Kwale County, Kenya (*n* = 231)

Of those respondents not currently using contraception, 231 (67%) reported that they intended to use in future. Among these, 108 (47%) reported not using since they were either currently pregnant or breastfeeding; 45 (20%) reported they were currently unmarried while 38 (17%) reported that they were currently either not having sex or having infrequent sex (Fig. 3). Further, 14 (6%) respondents reported not currently using contraception since they had not menstruated from their previous birth; 11 (5%) said that getting a child was up to God while 6 (3%) respondents said they were unable to conceive. An additional 28 (12%) respondents reported not currently using due to reasons that amounted to myths and misconceptions. A further 24 (11%) respondents reported current contraceptive non-use due to side effects or health-related concerns. Only 9 (4%) respondents reported that their non-use was due to religious prohibition or opposition by husband/male partner while 8 (4%) respondents reported being personally opposed to using contraception.

**Fig. 4:**
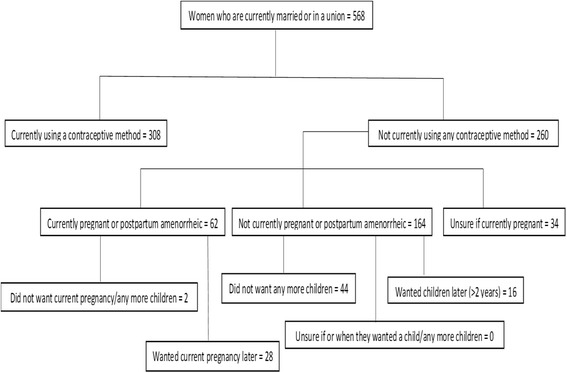
Bradley chart for unmet need among WRA from the Digo community residing in Kwale County, Kenya

We used the revised Bradley et al. algorithm for determining unmet need (Fig. 4) [25]. Of those respondents who were currently in a marital union, 46 (8%) were not using contraception despite reporting that they did not want any more children or that they were currently pregnant and had not desired their current pregnancy (unmet need for limiting). On the other hand, 44 (8%) respondents currently in a marital union were not using contraception despite reporting that they either wanted their current pregnancy later, had wanted their previous pregnancy later (>2 years) or were unsure if they wanted any more children (unmet need for spacing). In total, 90 (16%) respondents had an unmet need for contraception (total unmet need).

The odds of current contraceptive usage were higher among women who had ever attended school (aOR = 2.1, 95% CI: 1.4–3.4, *P* = 0.001); those who reported a previous birth (aOR = 5.0, 95% CI: 1.7–15.0, *P* = 0.004); those who reported attending ANC during their previous pregnancy (aOR = 4.0, 95% CI: 1.1–14.8, *P* = 0.04) as well as those who expressed intention to stop or delay future deliveries (aOR = 6.7, 95% CI: 3.3–13.8, *P* < 0.0001). These odds were adjusted for respondent’s age, educational level, marital status, history of a previous birth, ANC attendance in the previous birth and future fertility desire (Table 2).

**Table 2:** Determinants of current contraceptive usage among women from the Digo community residing in Kwale County, Kenya

	Univariate		Multivariate^a^	
	OR (95% CI)	*p*-value	OR (95% CI)	*p*-value
Respondent’s Age				
<29 years	Reference			
≥ 29 years	0.9 (0.7–1.2)	0.53		
School attendance				
Never attended school	Reference			
Ever attended school	1.8 (1.1–2.7)	0.01	2.1 (1.4–3.4)	0.001
Years of education				
< 8 years	Reference			
≥ 8 years	0.8 (0.6–1.2)	0.30		
ANC attendance				
No	Reference			
Yes	8.6 (2.5–29.3)	0.001	4.0 (1.1–14.8)	0.04
Ideal ANC attendance				
No	Reference			
Yes	1.1 (0.8–1.6)	0.48		
Ever given birth				
No	Reference			
Yes	4.5 (2.8–7.1)	<0.0001	5.0 (1.7–15.0)	0.004
Marital status				
Not in a marital union	Reference			
In a marital union	1.5 (1.1–2.1)	0.02	0.9 (0.6–1.4)	0.67
Not living with marital partner	Reference			
Living with marital partner	1.4 (0.9–2.1)	0.16		
Monogamous	Reference			
Polygamous	1.1 (0.7–1.7)	0.82		
Previous pregnancy intention				
Didn’t want to get pregnant	Reference			
Wanted to get pregnant	1.0 (0.7–1.3)	0.75		
Future fertility desire				
Prefer to have a child/another child soon	Reference			
No more children/Prefer to wait longer	7.5 (3.8–15.0)	<0.0001	6.7 (3.3–13.8)	<0.0001

^a^Adjusted for respondents’ age, educational level, marital status, ANC attendance, previous birth and future fertility desire

## Discussion

In this cross-sectional household survey of WRA from the Digo community residing in Kwale County, Kenya, we found that the levels of contraceptive usage and knowledge were higher than those previously reported in this setting. We also found correspondingly reduced levels of unmet need for contraception. A high proportion of our respondents confirmed previous, current and future intention to get pregnant. The proportion of those respondents who wanted to delay compared to those who wanted to limit further pregnancies was also high while majority of those who were currently not using contraception intended to use in future.

Additionally, our findings show that contraceptive usage in this setting is related to demand-side factors including educational attainment; history of a previous delivery; attendance of ANC services during previous pregnancy as well as expressed intention to stop or delay future childbirth. The proportion of respondents who reported not using contraception due to either method-related reasons, opposition to use (individual, spousal or religious) or lack of knowledge was significantly low. Majority of non-users gave fertility-related reasons for non-use.

The 2014 Kenya Demographic and Health Survey (KDHS) showed that 42% of currently married women in Kwale County aged 15–49 years were using any contraceptive method with only 38% currently using a modern method. Our study found a higher CPR of 54% for any contraceptive method which corresponds with recent findings that suggest a general decline in fertility in the coastal Kenya region [26]. Further, according to KDHS 2014, the most commonly used method in this setting was injectable Depo-Provera (22%) followed by contraceptive implants (7%). Our study reveals a similar pattern although the proportions are 2–3 times higher, with 45% of our respondents using injectable Depo-Provera and 23% using contraceptive implants.

Additionally, our findings reveal a total unmet need for contraception of 16% which is closer to the national average of 18% but lower than that previously reported for the wider Coast province (20.7%). This apparent decline in unmet need corresponds to the increase in CPR that we found and is also reflective of the general decline in unmet need for contraception registered nationally between 2008 and 2014. It is important to note however, that our findings focus only on those women who were in a marital union, who constituted more than three quarters of our respondents.

These findings are a reflection of the gains registered as a result of investments made by the County Government of Kwale with contributions from development partners in the health sector, towards improving uptake and utilization of contraceptive services [19–21]. The 2013 Kwale County Integrated Development Plan (CIDP) identifies population control as an important catalyst for economic development [15]. In this regard, the CIDP recommends that in order to achieve sustainable economic development, county resources should be dedicated to vigorous campaigns among WRA so as to encourage uptake and utilization of contraceptive services as well as to further improve MCH outcomes.

To this end, the County Government of Kwale has leveraged opportunities provided in the national community health strategy [27] which has led to actively supporting the establishment and ensuring the functionality of community health units as well as finding innovative means to incentivize the role of community health volunteers. This is aimed at strengthening community-based and community-led systems so as to encourage an enabling environment to ensure uptake and utilization of reproductive health services, including contraception. In this regard, the county government committed to spending nearly US$10 million over five years (2013–2018) [15].

It is instructive that in addition to enhancing supply-side interventions like improved supply chains for commodities as well as capacity improvement for healthcare providers, investments by the County Government of Kwale have focused on building up the demand for reproductive health services, including contraception. Several studies have shown that demand-side factors are as important as those on the supply-side in the delivery of MCH services [23]. For example, socio-cultural norms and gender-stereotypic dynamics have been shown to influence SRH-related attitudes and behaviors, especially regarding pregnancy intention and related uptake of contraception [22, 24].

Related to this, our findings reveal that even with high levels of current contraceptive usage, a large proportion of respondents did actually intend to get pregnant at the time that they did. Further, current contraceptive usage was mainly for delaying as opposed to limiting further pregnancies. This finding is consistent with studies that show that women in sub-Saharan Africa do in fact tailor their contraceptive usage to their current fertility desires [28, 29]. It reveals the need for contraceptive programs to target their approach so as to balance between encouraging the prevention of unwanted pregnancies versus enabling women safely achieve their fertility goals. This is further reflected in our finding that those respondents who expressed their intention to limit or space future pregnancies were nearly seven times as likely to be currently using a contraceptive method compared to those who did not.

Further, we found that women who reported having ever attended school were more than twice as likely to be currently using a contraceptive method. Several studies have shown that educated individuals are able to make better-informed health-related decisions including the choice to lower their fertility [30]. In sub-Saharan Africa in particular, completion of primary education among girls has been strongly associated with reduced fertility as well as a host of other predictors of socio-economic development of women [31, 32]. We also found that respondents who reported attending ANC during their previous or current pregnancy were four times more likely to be using a contraceptive method. Integration of contraception with other MCH services is important for maximizing women’s reproductive health outcomes since it allows for the quality of both services to be streamlined and improved to achieve optimal impact [33].

These findings need to be interpreted within the context of several limitations. First, this was a cross-sectional study and we are therefore, unable to infer causality. However, it does provide the basis for more formal assessment of any potential relationships observed. Secondly, we limited our respondents to women aged 18–45 years which was different from the age group of 15–49 years typically covered by demographic and health surveys. As a result, our findings may not be comparable on a one-to-one basis with previous KDHS findings in this setting. Our choice of age group was influenced by ethical considerations regarding the official age of consent in Kenya. We did allow however, emancipated minors to participate in the survey if they provided written informed consent. All other study procedures reflected those of a typical KDHS study.

## Conclusions

We found high levels of contraceptive utilization among WRA from a predominantly rural, Muslim community residing in coastal Kenya. These findings reflect the need for contraceptive programs to tailor their interventions with a focus on demand-side factors including understanding the fertility needs of users; ensuring educational achievement for girls and promotion of ANC attendance and skilled birth delivery. Further, our findings highlight the need for these contraceptive programs to develop interventions that are culturally-sensitive and locally-responsive so as to enhance contraceptive uptake and utilization.

## Additional file

Additional file 1: Sexual and reproductive health questionnaire. Questionnaire administered to all respondents within their households. (PDF 251 kb)

## Abbreviations

ANC: Antenatal care
CI: Confidence Interval
CIDP: County Integrated Development Plan
CPR: Contraceptive Prevalence Rate
IQR: Interquartile Range
IUCD: Intrauterine Contraceptive Device
KDHS: Kenya Demographic and Health Survey
LAM: Lactational Amenorrhea
MCH: Maternal Child Health
MNM: *Mama Na Mtoto* Project
MOMI: *Missed Opportunities in Maternal and Infant Health* Project
OCP: Oral Contraceptive Pills
OR: Odds Ratio
SDGs: Sustainable Development Goals
SRH: Sexual and Reproductive Health
TFR: Total Fertility Rate
WRA: Women of Reproductive Age
