# Supplementary material for: Pattern and determinants of contraceptive usage among women of reproductive age from the Digo community residing in Kwale, Kenya: results from a cross-sectional household survey
Source: BMC Womens Health. 2018 Jan 8;18:10. doi: 10.1186/s12905-017-0497-5 (PMC5759252; doi:10.1186/s12905-017-0497-5)
Supplement: Additional file 1: — Sexual and reproductive health questionnaire. Questionnaire administered to all respondents within their households. (PDF 251 kb) [file 12905_2017_497_MOESM1_ESM.pdf]

SEXUAL AND REPRODUCTIVE HEALTH SURVEY
QUESTIONNAIRE

Interviewer**: Thank you for taking time to answer the following questions. I ask that you provide accurate answers to the best of your ability. You are free not to answer any question that may be uncomfortable to you.**

Name of village: ____________________ Interviewer: ____________________

1. In what month and year were you born?

☐☐Month ☐Don't know month

☐☐☐☐Year ☐Don't know year

2. How old were you at your last birthday? ☐☐ *Completed years* ☐Don't know

3. Have you ever attended school? ☐Yes ☐ No, *skip to question 5*

4. How many years did you attend school? ☐☐*Completed years*

5. Have you ever given birth? ☐Yes ☐ No, *skip to question 12*
6. Do you have any sons/daughters to whom you have given birth who are now living with you?

☐Yes ☐ No, *skip to question 9*

7. How many sons live with you? ☐☐*Sons living at home*

8. How many daughters live with you? ☐☐*Daughters living at home*
9. Do you have any sons/daughters to whom you have given birth, are alive but don't live with you?

☐Yes ☐ No, *skip to question 12*

10. How many sons are alive but do not live with you? ☐☐*Sons living elsewhere*

11. How many daughters are alive but do not live with you? ☐ ☐ *Daughters living elsewhere*

12. Have you ever given birth to a boy or girl who was born alive but later died?

    ☐ Yes ☐ No, *skip to question 15*

13. How many boys dead? ☐ ☐ *Number of boys dead*

14. How many girls dead? ☐ ☐ *Number of girls dead*

15. Are you pregnant now? ☐ Yes ☐ No, *skip to question 17* ☐ Not sure, *skip to question 17*

16. How many months pregnant are you? ☐ ☐ *Number of completed pregnancy months*

17. Have you ever had a pregnancy that miscarried, was aborted or ended in a stillbirth?

    ☐ Yes ☐ No, *skip to question 20*

18. When did the last such pregnancy end?

    ☐ ☐ Month ☐ Don't know month

    ☐ ☐ ☐ ☐ Year ☐ Don't know year

19. How many months pregnant were you when the last such pregnancy ended?

    ☐ ☐ *Completed months* ☐ Don't know

20. When did your last menstrual period start?

    ☐ ☐ Day ☐ ☐ Month ☐ ☐ ☐ ☐ Year

    OR

    ☐ ☐ Days ago ☐ ☐ Weeks ago ☐ ☐ Months ago ☐ ☐ Years ago

OR

☐ Menopausal/has had hysterectomy ☐ Before last birth ☐ Never menstruated

21. From one menstrual period to the next, are there certain days when a woman is more likely to become pregnant?

☐ Yes ☐ No, *skip to question 23* ☐ Don't know, *skip to question 23*

22. Is this time just before her period begins, during her period, right after her period has ended or halfway between two periods? *(Tick all that apply)*

☐ Just before ☐ During ☐ Right after ☐ Halfway ☐ Other, specify ____________________

23. Now I would like to ask you about the various ways that a couple can use to delay or avoid a pregnancy. Have you ever heard of the following family planning methods?

- Female sterilization (women can have an operation to avoid having any more children)

  ☐ Yes ☐ No

- Male sterilization (men can have an operation to avoid having any more children)

  ☐ Yes ☐ No

- IUCD (women can have a loop or coil placed inside them to avoid having any more children)

  ☐ Yes ☐ No

- Injectable (women can have an injection that stops them from becoming pregnant)

  ☐ Yes ☐ No

- Implants (women can have one or more small rods placed under the skin in their upper arm which can prevent them from becoming pregnant)

  ☐ Yes ☐ No

- Pills (women can take a pill everyday to avoid becoming pregnant)

  ☐ Yes ☐ No

- Male condom (men can put a rubber sheath over their penis before sexual intercourse)

  ☐ Yes ☐ No

- Female condom (women can place a sheath in their vagina before sexual intercourse)

☐ Yes ☐ No

- Lactational amenorrhea method (women who are breastfeeding exclusively can avoid getting pregnant)

☐ Yes ☐ No

- Rhythm method (women do not have sexual intercourse on the days of the month they think they can get pregnant)

☐ Yes ☐ No

- Withdrawal method (men can be careful and pull out before climax)

☐ Yes ☐ No

- Emergency contraception (within 3 days after having unprotected intercourse, women can take special pills to prevent pregnancy as an emergency measure)

☐ Yes ☐ No

- Have you heard of any other ways or methods that women or men can use to avoid pregnancy?

☐ Yes, specify ____________________ ☐ No

24. Now I want to ask you questions about your current or last complete pregnancy. *For women who have never gotten pregnant, skip to question 62*. When you got pregnant, did you want to get pregnant at that time?

☐ Yes, *skip to question 27* ☐ No

25. If no, did you want to get a baby later on or did you not want any (more) children?

☐ Later ☐ No more, *skip to Q27*

26. If you wanted to have your baby later, how much longer did you want to wait?

☐☐ Months ☐☐ Years ☐ I don't know

27. Did you see/have you seen anyone for antenatal care of this pregnancy?

☐ Yes ☐ No, *skip to question 39*

28. If you did/have, whom did you see?

☐ Health worker ☐ TBA ☐ CHW ☐ Other, specify ____________________

29. How many months pregnant were you when you first received antenatal care during this pregnancy?

☐ ☐ Months ☐ I don't know

30. How many times did you receive/have you received antenatal care during this pregnancy?

☐ ☐ Number of times ☐ I don't know

31. As part of your antenatal care during this pregnancy, were any of the following done at least once?
    - Was your blood pressure measured?

      ☐ Yes ☐ No ☐ I don't know

    - Did you give a urine sample?

      ☐ Yes ☐ No ☐ I don't know

    - Did you give a blood sample?

      ☐ Yes ☐ No ☐ I don't know

32. During this pregnancy, were you told about the things to look out for that might suggest problems with the pregnancy?

☐ Yes ☐ No ☐ I don't know

33. During this pregnancy, were you given an injection in the arm to prevent the baby from getting tetanus, that is, convulsions after birth?

☐ Yes ☐ No, *skip to Q35* ☐ I don't know, *skip to Q35*

34. If you received the tetanus injection, how many times during the pregnancy did you get it?

☐ ☐ Number of times ☐ I don't know

35. During this pregnancy, were you given or did you buy any tablets or syrup to increase your blood levels?

☐ Yes ☐ No ☐ I don't know

36. During this pregnancy, were you given or did you buy any drugs for intestinal worms?

☐ Yes ☐ No ☐ I don't know

37. During this pregnancy, were you given or did you buy any drugs to keep you from getting malaria?

☐ Yes ☐ No, *skip to Q39* ☐ I don't know, *skip to Q39*

38. How many times during this pregnancy did you take the drugs to prevent malaria?

☐ ☐ Number of times ☐ I don't know

39. Where did you give birth during your last pregnancy? *If no previous pregnancy, skip to Q62*

☐ My home ☐ Other home ☐ Health facility ☐ Other, specify ____________________

40. Who assisted with the delivery?

☐ No one assisted ☐ Health worker ☐ TBA ☐ CHW ☐ Other, specify ____________________

41. How long after delivery did you stay there?

☐ ☐ Hours ☐ ☐ Days ☐ ☐ Weeks ☐ I don't know

42. Regarding your last delivery, was your child delivered by caesarean section, that is, did they cut your belly open to take out the baby?

☐ Yes ☐ No

43. Regarding your last delivery, was your child very large, larger than average, average, smaller than average or very small?

☐ Very large ☐ Larger than average ☐ Average ☐ Smaller than average ☐ Very small

44. Was the child weighed?

☐ Yes ☐ No, *skip to question 46* ☐ I don't know, *skip to question 46*

45. If the child was weighed, what was their weight?

☐ kilograms ☐ ☐ ☐ ☐ Milligrams ☐ I don't know

46. I would now like to ask you about checks on your health after delivery, for example, someone asking you questions about your health or examining you. Did anyone check on your health after you gave birth?

☐ Yes ☐ No, *skip to question 50*

47. Who checked on your health at that time?

☐ Health worker ☐ TBA ☐ CHW ☐ Other, specify ____________________

48. How long after giving birth did the first check take place?

☐ ☐ Minutes ☐ ☐ Hours ☐ ☐ Days ☐ ☐ Weeks ☐ I don't know

49. Where did this check occur?

☐ My home ☐ Other home ☐ Health facility ☐ Other, specify ____________________

50. I would like to ask you about checks on your baby's health after delivery, for example, someone asking you questions about their health or examining them. Did anyone check on the health of your baby after giving birth?

☐ Yes ☐ No, *skip to question 54*

51. Who checked on the health of your baby at the time?

☐ Health worker ☐ TBA ☐ CHW ☐ Other, specify ____________________

52. How long after delivery did the first check take place?

☐ ☐ Minutes ☐ ☐ Hours ☐ ☐ Days ☐ ☐ Weeks ☐ I don't know

53. Where did this check occur?

☐ My home ☐ Other home ☐ Health facility ☐ Other, specify ____________________

54. Regarding your last delivery, did you ever breastfeed your baby?

☐ Yes ☐ No, *skip to question 58*

55. How long after delivery did you put your baby to the breast?

☐☐ Minutes ☐☐ Hours ☐☐ Days ☐☐ Weeks

56. In the first three days after delivery, was the child given anything else to drink apart from breast milk?

☐ Yes ☐ No, *skip to question 58*

57. If yes, what else was the child given to drink?

☐ Water ☐ Cow's milk ☐ Other, specify ____________________

58. Has your baby ever been given childhood vaccinations?

☐ Yes ☐ No, *skip to question 62*

59. Do you have a card/booklet where your baby's vaccinations are written down? If yes, can I see it?

☐ Yes (seen) ☐ Yes (not seen), *skip to question 61* ☐ No, *skip to question 61*

60. Verify dates for all vaccinations received?

☐ All vaccinations received on schedule ☐ Vaccinations not received on schedule

61. Has your child received any vaccinations that are not recorded on this card/booklet, including those given during a national immunization campaign?

☐ Yes ☐ No

62. I would now like to ask you questions regarding your marital status. Are you currently married or living together with a man as if married?

☐ Yes, currently married ☐ Yes, living with a man ☐ Not in a union, *skip to question 68*

63. Is your husband/partner living with you now or is he staying elsewhere?

☐ Living with me ☐ Staying elsewhere

64. Does your husband have other wives or does he live with other women as if married?

☐ Yes ☐ No, *skip to question 67* ☐ I don't know, *skip to question 67*

65. If yes, in total including yourself, how many wives or live-in partners does your husband have?

☐ ☐ Total number ☐ I don't know

66. Are you the first, second……….wife? ☐ ☐ *Rank*

67. How old were you when you started living with your husband/partner?

☐ ☐ *Completed years* ☐ I don't know

68. If not currently married, have you ever been married or lived together with a man as if married?

☐ Yes, formerly married ☐ Yes, lived with a man ☐ Never, *skip to question 70*

69. If yes, what is your current marital status?

☐ Widowed ☐ Divorced ☐ Separated

70. Now I would like to ask you questions about sexual activity so as to get a better understanding of some important life issues. Let me assure you again that your answers are completely confidential and will not be told to anyone. If we should come to any question that you don't want to answer, just let me know and we will go to the next question. How old were you when you had sexual intercourse for the very first time?

☐ ☐ *Completed years* ☐ I don't know ☐ Not yet, *skip to question 78*

71. I would like to ask you some questions about your recent sexual activity. When was the last time you had sexual intercourse?

☐ ☐ Minutes ago ☐ ☐ Hours ago ☐ ☐ Days ago ☐ ☐ Weeks ago

☐ ☐ Months ago ☐ ☐ Years ago

72. What was your relationship to this person with whom you last had sexual intercourse?

☐ Husband/Partner ☐ Boyfriend not live-in ☐ Casual acquaintance ☐ Other, specify ____________

73. Did you use a condom the last time you had sexual intercourse?

☐ Yes ☐ No

74. How old was the person with whom you last had sexual intercourse?

☐ ☐ *Completed years* ☐ I don't know

75. Apart from this person, have you had sexual intercourse with any other person in the past 12 months?

☐ Yes ☐ No, *skip to question 77*

76. In total, how many different people have you had sexual intercourse with in the last 12 months?

☐ ☐ *Number of partners last 12 months* ☐ I don't know

77. In total, how many different people have you had sexual intercourse with in your lifetime?

☐ ☐ *Number of lifetime partners* ☐ I don't know

78. Have you ever been forced to have sexual intercourse against your will?

☐ Yes ☐ No

79. Do you know of a place where a person can get male condoms?

☐ Yes ☐ No, *skip to question 81*

80. If you wanted to, could you yourself get a male condom?

☐ Yes ☐ No ☐ I don't know/Not sure

81. Do you know of a place where a person can get female condoms?

☐ Yes ☐ No, *skip to question 83*

82. If you wanted to, could you yourself get a female condom?

☐ Yes ☐ No ☐ I don't know/Not sure

83. *For women who are menopausal, sterilized or unable to bear children, skip to Q88*. I have some questions about the future, would you like to have a child/another child or would you prefer not to have any more children?

☐ Prefer a child/another child ☐ No more, *skip to Q85* ☐ I don't know/Not sure, *skip to Q85*

84. How long would you like to wait from now before giving birth to a child/another child?

☐ ☐ Months ☐ ☐ Years ☐ Soon/Now ☐ I don't know

85. Are you currently using a family planning method?

☐ Yes, *skip to question 88* ☐ No

86. You have said you do not want another child soon/do not want any more children, can you tell me why you are not using a method to prevent pregnancy? *(Tick all that apply)*

Fertility-related reasons

☐ Not married ☐ Not having sex ☐ Infrequent sex ☐ Menopausal/Hysterectomy

☐ Can't get pregnant ☐ Currently pregnant/breastfeeding ☐ Not menstruated since last delivery

☐ It's up to God/Fatalistic

Opposition to use

☐ Respondent opposed ☐ Husband/partner opposed ☐ Others opposed

☐ Religious prohibition

Lack of knowledge

☐ Knows no contraceptive method ☐ Knows no source of contraceptive method

Method-related reasons

☐ Side effects/health concerns ☐ Lack of access/Too far ☐ Costs too much

☐ Preferred method not available ☐ No method available ☐ Inconvenient to use

☐ Interferes with body's normal processes ☐ Other, specify ____________________

87. If not currently using a contraceptive method, do you think you will use a contraceptive method to delay or avoid pregnancy at any time in the future?

☐ Yes, skip *to Q89* ☐ No, *skip to Q89*

88. Which family planning method are you currently using? *(Tick all that apply)*

☐ Female sterilization ☐ Male sterilization ☐ IUCD ☐ Injectable ☐ Implant

☐ Pills ☐ Male condom ☐ Female condom ☐ Lactational amenorrhea

☐ Rhythm method ☐ Withdrawal method ☐ Emergency contraception

89. If you could choose exactly the number of children to have in your whole life or if you could go back to the time you did not have any children and could choose exactly the number of children to have in your whole life, how many would that be?

☐ None ☐ ☐ Number of children

90. *If currently without husband/partner, skip to Q96*. Would you say that using a family planning method is mainly your decision, mainly your husband's decision or you both decide together?

☐ Respondent's ☐ Husband/partner's ☐ Joint decision ☐ Other, specify ____________________

91. Does your husband/partner want the same number of children that you want, or does he want more or fewer than you want?

☐ Same number ☐ More ☐ Fewer ☐ Don't know

92. How old was your husband/partner on his last birthday?

☐ ☐ *Completed years* ☐ I don't know

93. Did your husband/partner ever attend school?

☐ Yes ☐ No, *skip to question 95* ☐ I don't know, *skip to question 95*

94. How many years of school did he attend?

☐ ☐ *Completed years* ☐ I don't know

95. What kind of work does your husband/partner currently do?

☐ Self-employed ☐ Employed ☐ I don't know

96. As you know, some women take up jobs for which they are paid in cash or in kind. Others sell things, have a small business or work on the family shamba or family business. In the last seven days, aside from your own housework, have you done any of these things?

☐ Yes, *skip to question 98* ☐ No

97. If no, have you done any work in the last 12 months? ☐ Yes ☐ No, *skip to question 99*

98. *If currently without husband/partner, skip to Q101.* Who usually decides how your earnings will be used? You, your husband/partner or you and your husband/partner decide together?

☐ Respondent ☐ Husband/partner ☐ Joint decision ☐ Other, specify ________________

99. *If currently without husband/partner, skip to Q101.* Who usually makes decisions about healthcare for yourself? You, your husband/partner or you and your husband/partner decide together?

☐ Respondent ☐ Husband/partner ☐ Joint decision ☐ Other, specify ________________

100. *If currently without husband/partner, skip to Q101.* Who usually makes decisions about visits to your family or relatives? You, your husband/partner or you and your husband/partner decide together?

☐ Respondent ☐ Husband/partner ☐ Joint decision ☐ Other, specify ________________

101. In your opinion, is a husband justified in hitting or beating his wife in the following situations?

- If she neglects the children? ☐ Yes ☐ No ☐ Don't know
- If she argues with him? ☐ Yes ☐ No ☐ Don't know
- If she refuses to have sex with him? ☐ Yes ☐ No ☐ Don't know

We have come to the end of this questionnaire. I would like to thank you very much for your participation and assure you that all the answers you provided will be treated with confidentiality. No response you provided today will be linked directly to your name.
